# BaTiO_3_ Nanocarriers: Advancing Targeted Therapies with Smart Drug Release

**DOI:** 10.3390/pharmaceutics17091203

**Published:** 2025-09-16

**Authors:** Milica Ćurčić, Branka Hadžić, Martina Gilić, Zorica Lazarević, Andjelija Ilić

**Affiliations:** Institute of Physics Belgrade, University of Belgrade, Pregrevica 118, 11080 Belgrade, Serbia; branka@ipb.ac.rs (B.H.); martina@ipb.ac.rs (M.G.); lzorica@ipb.ac.rs (Z.L.)

**Keywords:** barium titanate (BaTiO_3_), drug delivery, biocompatibility, nanoparticle carriers, biomedical applications

## Abstract

**Background/Objectives**: Barium titanate (BaTiO_3_)-based nanocarriers have emerged as versatile and promising platforms for targeted drug delivery, owing to their unique combination of biocompatibility, piezoelectric and ferroelectric properties, as well as responsiveness to external stimuli. These multifunctional ceramic nanoparticles can be precisely engineered to enable spatiotemporally controlled release of therapeutic agents, triggered by physical stimuli such as ultrasound, light, magnetic fields, temperature changes, and pH variations. Such an approach enhances treatment efficacy while reducing systemic side effects. **Methods**: This review provides a comprehensive overview of the latest advancements in the development and biomedical application of BaTiO_3_-based nanocarriers. Special emphasis is placed on modern synthesis strategies, surface functionalization methods, and the integration of BaTiO_3_ with other functional nanomaterials to create hybrid systems with improved therapeutic performance. Key challenges in clinical translation are also discussed, including biocompatibility assessment, biodistribution, and regulatory requirements. **Conclusions**: BaTiO_3_-based nanocarriers show promise as materials well suited for advanced biomedical applications. The paper concludes with an outline of future research directions aimed at optimizing these advanced nanosystems for precision and personalized medicine, with applications in oncology, anti-infective therapy, and regenerative medicine.

## 1. Introduction

Since its discovery in the 1940s, barium titanate (BaTiO_3_) has held a prominent position in the field of material science and electronic engineering. As the first ceramic material to exhibit ferroelectric behavior at room temperature, BaTiO_3_ has been widely adopted in the development of capacitors, thermistors, actuators, and piezoelectric sensors [1,2]. Its exceptionally high dielectric constant, combined with piezoelectric and pyroelectric properties, made it an indispensable component in a wide range of industrial and consumer electronics. For decades, BaTiO_3_ was primarily utilized in passive devices, serving as a reliable and tunable insulating material in multilayer ceramic capacitors and electro-optical modulators [3,4,5].

However, the evolution of nanotechnology as well as biomedical engineering has revealed the untapped potential of BaTiO_3_ as a multifunctional, stimulus-responsive nanomaterial, which could be employed in diagnostic and therapeutic applications. BaTiO_3_ exhibits distinct physical behaviors, including enhanced surface activity, tunable ferroelectric domains, and increased responsiveness to external stimuli such as mechanical stress or temperature gradients, when synthesized in the nanoscale regime. These characteristics have catalyzed its transformation from an industrial dielectric to an active biomedical nano platform [6,7,8]. Currently, the wide array of nanomaterials is being proposed for drug delivery and nanomedicine, including liposomes, polymeric micelles, dendrimers, and mesoporous silica; however, BaTiO_3_ offers certain unique advantages. As a ferroelectric and piezoelectric nanocarrier, it can actively respond to physical stimuli such as ultrasound, modified pressure and temperature, thus enabling externally controlled spatiotemporal drug release [9,10]. In contrast to the traditional carriers, typically relying on passive mechanisms like diffusion or degradation, BaTiO_3_ nanoparticles (BTNPs) are capable of electrical stimulation, mechanical sensing, and bioelectrical interactions.

Moreover, the use of BaTiO_3_ in targeted delivery systems is also supported by its surface modifiability and colloidal stability. Namely, the surface of BaTiO_3_ can be functionalized with biorecognition elements (e.g., antibodies, peptides, aptamers) to direct these nanocarriers toward specific cells or tissues, such as tumor microenvironments or inflamed regions. The same particles can also be engineered to respond to the local pH, enzyme activity, oxidative stress, or electromagnetic fields, making them powerful agents in personalized and precision medicine [7,11,12]. Further, recent studies have explored the possible role of BaTiO_3_ as theranostic nano platforms, which use a single nanosystem to carry out the combined diagnostics and therapy. For instance, BTNPs stimulated by the applied ultrasound can simultaneously release chemotherapeutic drugs and generate reactive oxygen species (ROS) for sonodynamic therapy (SDT), effectively killing cancer cells through both biochemical and mechanical actions. BaTiO_3_-based systems integrated with photothermal, magnetic, or photoacoustic components, can enable multimodal treatment consisting of combined real-time imaging, active targeting, and synergistic therapeutic actions [13,14,15,16,17].

The favorable biocompatibility of BaTiO_3_ has been demonstrated in a variety of in vitro and in vivo biological models. Recent studies report minimal inflammatory responses, low systemic toxicity, and prolonged circulation times with the appropriately surface- modified nanoparticles (e.g., via PEGylation, lipid coating, or natural polysaccharides). Chemical inertness, mechanical robustness, and resistance to biodegradation of BaTiO_3_ support its integration into long-term biomedical applications, such as implantable drug release systems or bioelectric scaffolds [17,18,19,20].

Despite the mentioned benefits, there are also multiple challenges obstructing the translation of BaTiO_3_ nanocarriers into the medical practice. Firstly, more studies is needed on the problems of their biodistribution and clearance, in particular investigating possible accumulation in organs such as the liver or spleen. Long-term toxicity needs to be much more investigated, especially in relation to the degradation of byproducts or possible ion release. Scalability and reproducibility issues are present, which are critical to resolve prior to any industrial manufacturing and clinical-grade standardization [21,22]. In connection with that, regulatory classification would have to be performed, as the inorganic nanomaterials typically fall outside the traditional pharmaceutical frameworks. Still, the use of BTNPs seems realistic, provided that the above problems can be resolved. Many innovations in nanoparticle synthesis are being proposed, as well as the new methods of surface engineering, biofunctionalization, and hybrid material development. Currently, the incorporation of machine learning (ML) and AI-driven modeling tools in materials research allows for the prediction and optimization of parameters such as particle size, chemical potentials, and estimates of stimuli-response behavior. These new methods can help in accelerating preclinical development and translational design [23,24].

The most significant novelty brought by the BaTiO_3_ nanocarriers, in comparison with the other drug delivery platforms, is the combination of many advantageous properties in a single material, along with a multitude of ways to trigger or induce the drug release. In that sense, it represents a truly innovative and multifunctional platform offering enhanced functionality as compared with the existing drug delivery systems (liposomes, polymeric carriers, or mesoporous silica). While many conventional platforms predominantly rely on passive release mechanisms, such as diffusion or polymer degradation, BaTiO_3_ nanocarriers possess a unique union of physicochemical properties, including piezoelectricity, ferroelectricity, and pyroelectricity. These features enable them to convert many types of external physical stimuli, including ultrasound, mechanical stress, or temperature gradients, into the localized electrical impulses and also allow precise spatiotemporal control of the planned drug release. Such an active mechanism constitutes a significant advancement over the passive systems. It is particularly of advantage for personalized therapy, where precise control over release kinetics is essential. In addition, BaTiO_3_ is a chemically stable and biocompatible inorganic material, allowing long-term use in biological environments without the degradation that often limits organic carriers. Its structure can be further tailored through doping with rare-earth ions (Ho^3+^, Er^3+^, Gd^3+^), which provides fine adjustment of electrical, magnetic, and optical properties and enables the development of theranostic systems that integrate diagnostic and therapeutic functions within a single nanosystem. Surface functionalization (e.g., PEG, SiO_2_) further improves biodistribution, reduces cytotoxic effects, and allows selective targeting of specific tissues or cells, thereby enhancing the precision and efficacy of therapy. Collectively, these attributes define BaTiO_3_ nanocarriers as a new generation of intelligent, actively responsive drug delivery systems, with the potential to overcome the limitations of conventional smart platforms and enable integrated theranostic solutions in nanomedicine.

Here we contribute a review providing a comprehensive summary on the use of BaTiO_3_ nanocarriers in advanced drug delivery, including the discussion on the scientific and technological basis supporting such use. This review covers the published literature from the early 2000s to early 2025, a period during which research on BaTiO_3_ nanomaterials expanded from fundamental studies of their ferroelectric and piezoelectric properties to biomedical investigations. Notably, most of the preclinical and medical studies have been conducted in the last decade (2015–2025), reflecting a growing interest in their applications as nanocarriers for drug delivery and theranostic platforms. The contents of this review can be summarized as follows. The Introduction section gives a relatively short summary of key points regarding this work. It is followed by the description of structural and functional properties of BaTiO_3_, making it an advantageous nanomaterial for biomedical applications. Descriptions are also presented in connection with planned use scenarios. Synthesis methods which can influence material properties are explained, as well as surface functionalization strategies, and suggested smart release mechanisms, i.e., the release in response to various stimuli. The subsequent Section 3 justifies the need for new nanocarrier platforms, presenting major proposed applications and main properties enabling those mentioned applications. Section 4 gives a critical comparison with conventional nanocarriers. Finally, we evaluate material biocompatibility, applications in the treatment of cancers, infections, and regenerative medicine, and the still existing barriers to clinical translation, in Section 5. We consider future perspectives of BaTiO_3_ in the field of personalized drug delivery. The Conclusion once again emphasizes the main findings that were presented in this work.

## 2. Structural Properties and Synthesis Methods of BaTiO_3_

Barium titanate (BaTiO_3_) is a prototypical ferroelectric oxide with a perovskite-type structure and a wide range of functional properties, including ferroelectricity, piezoelectricity, pyroelectricity, and a high dielectric constant. This unique combination of features is of advantage for its use in capacitors, sensors, actuators, and electro-optic devices, but also in biomedical systems and stimuli-responsive drug delivery systems.

**Figure 1:**
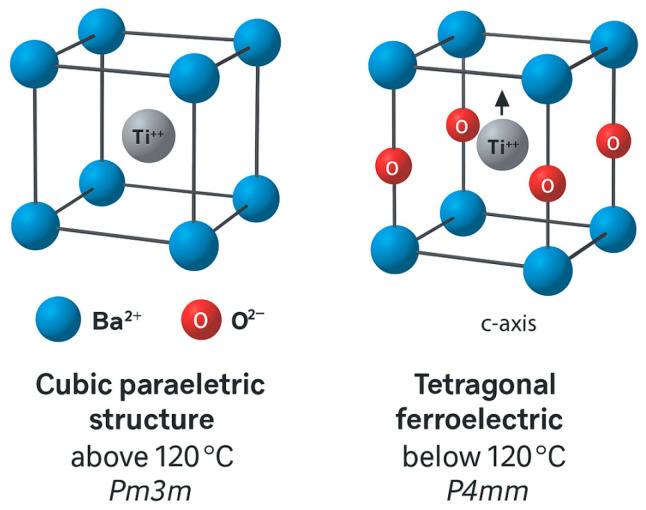
Crystallographic phase transition of BaTiO_3_: cubic paraelectric vs. tetragonal ferroelectric structure.

The perovskite ABO_3_ structure adopted by the BaTiO_3_, in which Ba^2+^ occupies the A-site and Ti^4+^ is centrally coordinated within an oxygen octahedron (please see Figure 1), allows for multiple phase transitions it can undergo. Its crystallographic symmetry is strongly temperature-dependent, and its functional performance significantly influenced by the series of occurring phase transitions. Namely, at temperatures above 120 °C, BaTiO_3_ exhibits a cubic, paraelectric structure (space group Pm3m). Upon cooling below this temperature, it transforms into a tetragonal ferroelectric phase (P4mm), characterized by the off-center displacement of Ti^4+^ ions along the c-axis and the emergence of spontaneous polarization (Figure 1) [25]. Further cooling induces orthorhombic (Amm2) and rhombohedral (R3m) ferroelectric phases, with polarization rotating accordingly along specific crystallographic axes [26].

The displacement of Ti^4+^ ions from the center of the octahedron leads to the formation of electric dipoles, the alignment and reorientation of which under an external electric field define the ferroelectric behavior of the material. The presence of these switchable dipoles results in a characteristic polarization–electric field hysteresis loop, making BaTiO_3_ a model ferroelectric used in non-volatile memory and field-tunable devices.

High dielectric permittivity of BaTiO_3_ peaks near the Curie temperature (120 °C) due to the contribution of domain wall motion and lattice polarization. This property underpins its use in multilayer ceramic capacitors (MLCCs), one of the most widespread passive components in modern electronics [27]. The BaTiO_3_ piezoelectric response, particularly prominent in the tetragonal phase, on the other side enables its employment in ultrasonic transducers, sensors, and energy harvesting devices [28], all making use of electrical polarization induced by the applied mechanical stress. The induced electrical polarization in response to the temperature changes, due to the surface charge generation caused by temperature fluctuations, i.e., the pyroelectricity of BaTiO_3_, is typically utilized in infrared detection and in thermally activated devices. Additionally, BaTiO_3_ exhibits high chemical and mechanical stability, which combined with the above mentioned properties make this material suitable for diverse electroactive applications.

**Figure 2:**
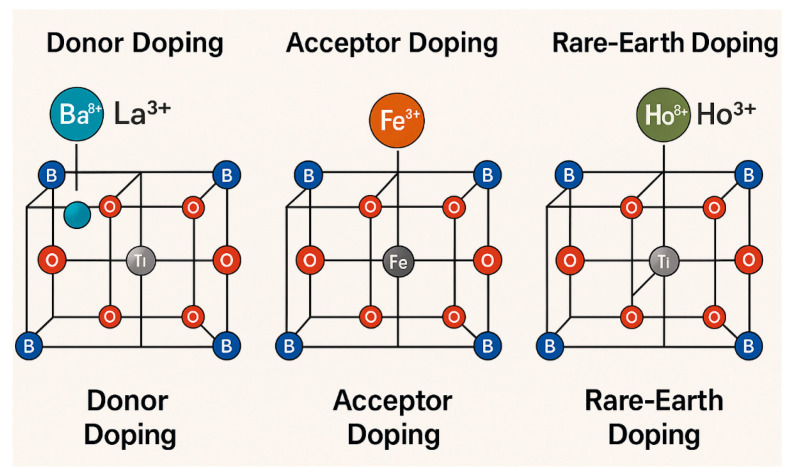
Dopant incorporation in BaTiO_3_: donor doping (La^3+^) at the A-site enhances dielectric properties; acceptor doping (Fe^3+^) at the B-site promotes domain wall pinning; rare-earth doping (Ho^3+^) introduces multifunctionality for biomedical applications.

Intrinsic point defects, particularly oxygen vacancies, play a critical role in modifying the electrical conductivity and ferroelectric properties of BaTiO_3_. Oxygen vacancies can create localized states within the bandgap, increase leakage currents, and destabilize ferroelectric domains [29]. To mitigate these effects, various doping strategies have been employed so far. For example, donor doping with La^3+^, Nb^5+^, or Y^3+^ reduces the concentration of oxygen vacancies and improves dielectric performance. Acceptor doping with Fe^3+^ or Mn^2+^ alters the defect chemistry to favor domain wall pinning, enhancing fatigue resistance. Rare-earth doping, e.g., Ho^3+^, Er^3+^, introduces multifunctionality, such as upconversion luminescence and magnetic interactions, expanding the applicability of BaTiO_3_ in biomedical imaging and theranostics (Figure 2) [30,31]. Surface functionalization and chemical tailoring further enable modulation of surface charge (zeta potential), as well as colloidal stability and interaction with biological interfaces, making BaTiO_3_ increasingly adaptable for biointegration.

The choice of synthesis method is highly important, as it must balance phase stability, surface chemistry, and particle uniformity depending on the target application, especially when integrating BaTiO_3_ into hybrid nanocomposites, or when adapting its properties for interaction with the biological systems. The synthesis method is known to affect the size, morphology, crystallinity, and phase purity of BaTiO_3_, all of which directly influence its functional properties. Among the various synthesis routes, the sol–gel method offers excellent control over stoichiometry and homogeneity at relatively low temperatures, producing fine particles with narrow size distributions [32]. Microwave-assisted hydrothermal synthesis combines the benefits of aqueous processing with rapid and uniform volumetric heating, thereby enabling the formation of structures such as the well-defined, noncentrosymmetric BaTiO_3_ nanocubes with controlled morphology and high crystallinity, described in [33]. Microwave-assisted methods yield nanoparticles with reduced grain size and improved dielectric properties, which is suitable for advanced charge storage (electronic applications) and potentially also for bio-integrated devices. Other techniques include solid-state reactions, industrially scalable but limited by coarse grain sizes, and combustion and co-precipitation methods, offering faster synthesis and high throughputs.

## 3. The Need for Targeted Drug Delivery and Biomedical Relevance of BaTiO_3_ in the Context of Smart Drug Delivery

**Figure 3:**
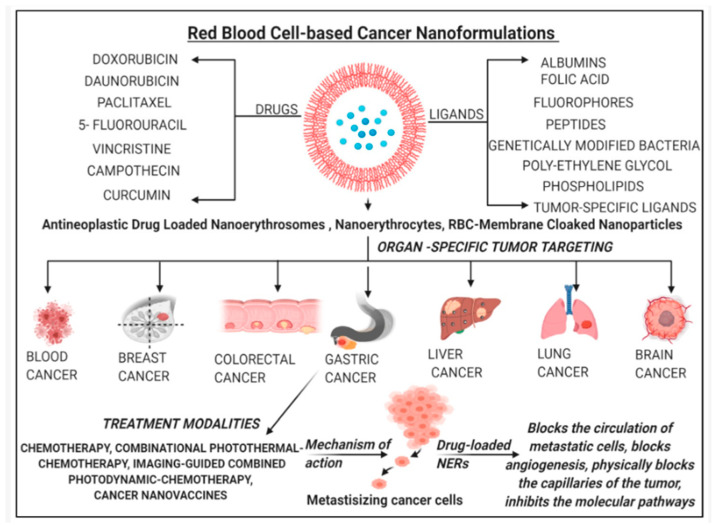
Summary of red blood cell (RBC)-based anticancer nanoformulations (anticancer-loaded- NERs) along with their mechanisms of action, reproduced from [34], licenced under a Creative Commons Attribution.

Targeted drug delivery is an advanced therapeutic strategy increasingly used nowadays, which is designed to bring pharmaceutical agents directly to diseased tissues, thereby improving treatment efficacy while minimizing damage to the healthy cells. Such approaches are crucial in oncology, where conventional chemotherapeutics often exhibit high systemic toxicity, short circulation half-life, and low tumor selectivity. By enabling the precise localization of drug release, targeted delivery systems reduce adverse effects and enhance therapeutic indices of anticancer agents. A transformative advancement in the field of nanomedicine was brought by the development of smart drug delivery systems (SDDS). These are the systems engineered to respond to the specific internal or external stimuli, thus enabling the spatiotemporal control over drug release. Unlike conventional drug delivery methods, which often result in systemic distribution and undesirable side effects, targeted drug delivery and in particular SDDS offer a possibility to deliver the therapeutic agents selectively to diseased tissues, while minimizing the exposure of healthy cells. A breadth of possibilities in targeting specific organs, delivering either drugs or ligands, and employing one or more of the possible mechanisms of action is illustrated in Figure 3, for the erythrocyte-based biomimetic SDDS [34]. The obtained level of precision is especially critical in the cancer treatment, where the off-target cytotoxicity significantly limits the efficacy of chemotherapy [34,35].

Among the various stimuli-responsive platforms developed to date, barium titanate (BaTiO_3_)-based nanomaterials stand out due to a multitude of controllable electromechanical, ferroelectric, piezoelectric, and pyroelectric properties, as well as their robust chemical stability and biocompatibility. Namely, the in vivo studies have demonstrated that BaTiO_3_ nanoparticles do not provoke significant immune responses or organ toxicity in animal models, provided that the appropriate surface modifications are applied [36]. In vitro cytotoxicity tests have confirmed their compatibility with various mammalian cell lines, including fibroblasts and epithelial cells. When integrated into the biocomposite scaffolds such as poly(ethylene oxide)/silk fibroin (PEO/SF), BaTiO_3_ has been shown to enhance cellular adhesion, proliferation, and viability, supporting its use in soft tissue engineering [37]. BTNPs can be engineered to respond to a variety of physiological and pathological triggers, including pH, temperature, enzymes, ultrasound, and electric fields. These multifaceted capabilities enable controlled, reversible, and targeted drug release, as required of the next-generation SDDS. The so far proposed use scenarios for BTNPs can roughly be divided into several groups according to the planned treatments and material properties mostly of interest for a particular application.

### 3.1. Antibacterial Applications of BaTiO_3_ (BTNPs)

The antibacterial effects of BaTiO_3_ arise from intrinsic physicochemical mechanisms as well as synergistic functionalities when combined with antibiotics or biomaterials. The main mechanisms of antibacterial action of BTNPs, impairing bacterial viability and adhesion, include: generation of reactive oxygen species (ROS), which induce oxidative stress and damage bacterial membranes; electrostatic interaction with negatively charged bacterial cell walls, leading to the damage or leakage of cellular content; mechanical disruption of bacterial adhesion and interference with biofilm formation; inhibition of microbial colonization on material surfaces [38,39]. The last property makes BaTiO_3_ highly suitable for incorporation into the biomedical polymers and coatings.

In a recent study [38], the multifunctional antibacterial potential of BTNPs was evaluated by studying their effect on the adhesion of *Staphylococcus epidermidis*. The authors have prepared maxillofacial silicone composites with varying concentrations of BTNPs and tested bacterial adhesion under the in vitro conditions. A concentration-dependent reduction in bacterial adhesion has been observed, with higher BTNPs loading leading to more decreased *S. epidermidis* colonization. Given a serious clinical challenge posed by the *Staphylococcus* biofilms, the antibacterial effects attributed to a combination of surface charge alterations, possible ROS generation, and the altered nano-topography, could be used to prevent the bacterial adhesion on implantable devices and prosthetic surfaces. Most importantly, the incorporation of BTNPs did not adversely affect the mechanical properties of the silicone. In an another study [39], a graphene–BaTiO_3_ (GBT) nanocomposite has been developed, which has combined the piezoelectric properties of BaTiO_3_ with the conductivity and high surface activity of graphene to create a multifunctional nanosystem responsive to both mechanical and light-based stimulation. While the BaTiO_3_ can induce ROS and oxidative stress damaging to bacterial cell membranes, proteins, and nucleic acids, when exposed to light, the GBT composite generated substantially increased levels of ROS, effectively killing both *Escherichia coli* and *Staphylococcus aureus*, under the white light irradiation. Beyond the antibacterial efficacy, the GBT nanocomposite also enhanced fibroblast proliferation and migration in vitro (NIH-3T3 cells), which are key processes in tissue regeneration.

Similarly, BTNPs embedded in polymer matrices have shown promising antimicrobial potential. In the study by Jaafar et al., BaTiO_3_ was incorporated into a PMMA/PEO polymer blend along with SiC to form multifunctional nanostructures. These nanohybrids exhibited significant antibacterial activity against both Gram-positive and Gram-negative bacteria, which has been attributed to the combined effects of the polymer matrix and the active inorganic components. Although the system has not been explicitly functionalized with conventional antibiotics, the observed antibacterial enhancement has suggested that BaTiO_3_-containing nanocomposites can disrupt bacterial integrity and interfere with cell viability. The study has also highlighted the synergistic interaction between the polymeric carriers and ceramic nanoparticles in amplifying antibacterial efficacy, emphasizing their potential role in addressing drug-resistant infections [40].

### 3.2. Tissue Regeneration and Wound Healing

The BaTiO_3_-based nanoscaffolds have emerged as promising platforms for delivering electromechanical stimulation in regenerative medicine. As mentioned, BaTiO_3_ can be integrated into the biocomposite scaffolds such as the PEO/SF, enhancing cellular adhesion, proliferation, and viability [37]. Additionally, materials engineered with the addition of BaTiO_3_ can employ the piezoelectric properties to successfully convert mechanical stresses into the localized electric signals, thereby mimicking the native bioelectrical environment of excitable tissues. Such functionality has been shown to enhance osteogenic differentiation by stimulating cell adhesion, proliferation, and maturation [41]. A recent study, [42], demonstrated the fabrication of 3D-printed piezoelectric nanocomposite scaffolds composed of BTNPs embedded in a polyhydroxybutyrate (PHB) matrix. Such scaffolds exhibited favorable mechanical integrity, sustained biodegradability, and stable piezoelectric performance, all of which contributed to improved osteoconductivity and cellular response. The findings underscore the potential of BaTiO_3_-based systems not only for bone regeneration but also as a foundational strategy for developing smart scaffolds in other load-bearing or electrically responsive tissues, such as cardiac or neural tissues.

The applications of BaTiO_3_ in regenerative medicine additionally benefit from the shown antibacterial effects, making this material a convenient choice for producing wound materials and antimicrobial coatings. For example, in a study of GBT nanocomposite aimed at combined mechanical and light-based stimulation [39], antibacterial effects were increased by generating significantly increased levels of ROS, effectively killing bacteria. At the same time, the GBT nanocomposite enhanced fibroblast proliferation and migration in vitro (NIH-3T3 cells), whereas the in vivo investigation confirmed its utility in wound healing. The in vivo experiments using infected wound models in mice showed accelerated wound closure, reduced inflammation, and enhanced collagen deposition in the GBT-treated group in comparison with the control, with no observable systemic toxicity, indicating strong biocompatibility and safety. These findings position BaTiO_3_-based materials, particularly in composite forms like in the GBT, as promising candidates for the development of advanced antimicrobial coatings, smart wound dressings, and multifunctional medical textiles. Currently, direct integration of BaTiO_3_ into fabrics remains a developing research avenue.

### 3.3. Use of Piezoelectric Properties for Controlled Drug Release

The intrinsic piezoelectric properties of BTNPs enable their use as the drug carriers responsive to the external stimuli, particularly in applications requiring precise spatial and temporal control of therapeutic release. Upon exposure to ultrasound or mechanical stress, BTNPs undergo deformation, generating localized electric fields at their surface. These fields can induce conformational changes or disrupt encapsulating matrices, resulting in the release of the drug payload directly at the targeted site. Therefore, the ultrasound-triggered release mechanism provides several clinical advantages. It allows for minimization of systemic drug exposure, thereby reducing side effects; spatial precision, by confining release to the target tissue; and temporal control, enabling dose scheduling based on disease progression or real-time patient needs. Most importantly, the application of ultrasound not only triggers the release but it can also increase cellular uptake by enhancing cell membrane permeability, further improving therapeutic efficacy. This dual function, drug liberation combined with the membrane permeabilization, has been shown to enhance drug delivery in oncological applications, where treatment resistance and localization remain major clinical challenges. For example, Marino et al. developed piezoelectric BaTiO_3_ nanostimulators which, when activated by ultrasound, generate localized electric fields capable of inhibiting the proliferation of glioblastoma multiforme cells [43]. The proposed strategy does not involve chemical agents, which is attractive for noninvasive targeted therapeutic approaches. It is particularly useful for the treatment of deep tissue tumors, which are hard to reach with the traditional systems. It is also a non-invasive alternative for the treatment of drug-resistant tumors, while also showing promise in managing chronic inflammatory diseases and localized infections, where the controlled, localized therapy is essential for avoiding recurrence or systemic toxicity [44].

Furthermore, targeting specificity can be improved by modifying BaTiO_3_ nanoparticles with surface coatings that enhance accumulation at the tumor location. For instance, Wang et al. demonstrated that ultrasmall BaTiO_3_ nanoparticles coated with DSPE-PEG preferentially accumulate in hypoxic tumor regions, where they can be activated by ultrasound to induce therapeutic effects via piezo catalysis and water splitting. This has led to the generation of reactive oxygen species (ROS) even in oxygen-deficient tumor microenvironments. Such functionalized nanocarriers minimize off-target distribution and improve therapeutic efficacy by exploiting tumor-specific microenvironmental conditions [45].

### 3.4. Catalytic Approaches in Smart Drug Release

One of the most widely exploited features of BaTiO_3_ in smart delivery platforms is its pH-responsiveness, by using the fact that the microenvironment of tumors is slightly acidic, with extracellular pH on the order of pH ≈ 6.5–6.8, in comparison with the normal physiological pH (~7.4). By functionalizing the surface of BaTiO_3_ with acid-labile linkers, pH-sensitive groups (e.g., imine or hydrazone bonds), or encapsulating pH-degradable polymers like poly(β-amino esters), or chitosan, BaTiO_3_-based systems can remain stable in circulation but rapidly degrade, disassemble, or swell in acidic environments (tumor regions), thereby releasing encapsulated drugs at the desired site [41,46,47,48]. This strategy improves tumor targeting specificity, minimizes systemic drug exposure, and reduces the likelihood of off-target toxicity. Moreover, composite systems combining BaTiO_3_ with hydrogels, microparticles, or biodegradable polymers can be tailored to provide dual responsiveness, e.g., pH + ultrasound or pH + temperature. Thereby, they enable multi-level control over the drug delivery kinetics. For example, a pH/ultrasound dual-triggered hydrogel embedded with BaTiO_3_ nanoparticles remains dormant under physiological conditions, but releases drugs rapidly in the acidic tumor site, when exposed to the ultrasound [49]. Enhanced drug encapsulation efficiency and increased accumulation in solid tumors, via the enhanced permeability and retention (EPR) effect, have been achieved by the use of pH-sensitive micelles incorporating BaTiO_3_ cores. A burst drug release under acidic conditions has been demonstrated for such systems [14]. It has been noted that such systems also facilitate endosomal escape, a critical step for intracellular drug delivery, as BaTiO_3_ nanoparticles have the ability to disrupt the acidic endosomal membrane via local electric field changes triggered by pH or ultrasound stimulation [50].

In addition to pH sensitivity, pyrocatalysis of BTNPs can be employed to enable the temperature-responsive design. The ability to generate or respond to heat is particularly valuable in hyperthermia-assisted cancer therapy, where tumors are locally heated (e.g., to 42–45 °C) in addition to chemotherapy or radiation. By co-engineering BTNPs with thermo-responsive polymers (e.g., poly(*N*-isopropyl acrylamide)), which undergo phase transitions at elevated temperatures, drug release could be further facilitated [51]. The pyroelectric property of BaTiO_3_ additionally enables the generation of transient surface charges upon thermal fluctuations, a phenomenon which can get enhanced through electric poling to significantly boost the overall catalytic performance. This behavior holds promise for biomedical applications, where temperature-induced dielectric interactions could be harnessed for controlled drug release or improved cellular interactions [52]. It is worth noting that, due to the BaTiO_3_ surface polarizability and cation-exchange capacity, it could act as a reservoir for charged therapeutic molecules, which could be released as needed through stimulus-induced desorption or degradation. For instance, coupling the BaTiO_3_ to enzyme-cleavable peptides or ROS-sensitive linkers, systems could be created which release their cargo only in the presence of tumor-associated enzymes (e.g., matrix metalloproteinases) or inflammatory signals [53].

### 3.5. Photothermal and Photodynamic Therapy Using BaTiO_3_

The BaTiO_3_-based nanoplatforms have also been considered as multifunctional agents in photothermal therapy (PTT) and photodynamic therapy (PDT). One of the particularly promising strategies involved the engineering of oxygen vacancies within the BaTiO_3_ lattice. So introduced structural defects modulate the electronic structure, narrowing the bandgap and enhancing light absorption, charge separation, and catalytic performance under irradiation. Such nanoparticles exhibited enhanced near-infrared (NIR) absorption of light and peroxidase-like activity. Effective ROS generation, photothermal conversion, and tumor inhibition when exposed to a single NIR laser were demonstrated, offering a one-step therapeutic modality for photothermal, photodynamic, and catalytic therapy Photocatalytic ROS generation occurs even under hypoxic conditions, overcoming one of the primary limitations of the traditional PDT [54]. The resulting therapy is efficient and non-invasive for the surrounding healthy tissues.

BaTiO_3_–ZnO heterostructures have produced markedly enhanced cytotoxic effects in comparison to their individual components, particularly at elevated concentrations. The synergistic combination of BaTiO_3_ and ZnO, especially when modified to introduce oxygen vacancies, has promoted the production of reactive oxygen species (ROS) under ultrasound stimulation and light irradiation. Composites resembling BaTiO_3_(H_2_)/ZnO_x_ exhibited the most pronounced reduction in tumor cell viability, particularly when co-administered with the hydrogen peroxide (H_2_O_2_), suggesting an amplified oxidative stress response [55]. These studies have highlighted the collaborative roles of oxygen vacancies and pyroelectric polarization in enhancing the photoelectrocatalytic activity of BaTiO_3_-based systems. The influence and mechanism of surface oxygen vacancies on the piezo-photocatalytic activity of BaTiO_3_ have been further studied in [56,57].

A study by Wang et al. [58], reported on the design of polyelectrolyte-coated BaTiO_3_ nanoparticles combining second-harmonic generation (SHG) for high-contrast bioimaging guidance with the ability to carry photosensitizers for precise photodynamic therapy (PDT). Positive surface charge of BTNPs resulted in enhanced internalization in cancer cells and attributed to stronger electrostatic interactions with the negatively charged cell membrane, ultimately improving the efficacy of the imaging-guided photodynamic therapy. Owing to their nonlinear optical and dielectric properties, BTNPs enabled simultaneous diagnostics and therapy (theranostics) within a single system [58].

### 3.6. Enhanced Cellular Uptake Through Surface Charge Modulation

The surface charge of BTNPs plays a critical role in their interactions with biological membranes and significantly influences the efficiency of cellular uptake. By modulating the zeta potential of these nanoparticles, their interaction with target cells can be enhanced, particularly through the introduction of positive surface charges, which have a stronger affinity for the negatively charged cell membranes characteristic of cancer cells and other diseased tissues [7]. Functionalization of BTNPs with cationic polymers such as polyethyleneimine (PEI) or chitosan has proven effective in shifting the surface charge towards the more positive values, enhancing electrostatic attraction and facilitating membrane adhesion and endocytosis. In one study, BTNPs coated with polyelectrolyte layers exhibited significantly enhanced stability and higher cellular internalization, supporting their utility in drug delivery applications [58]. Similarly, chitosan-coated BaTiO_3_ nanoparticles demonstrated reduced cytotoxicity and improved dispersion, further confirming their biocompatibility and suitability for in vivo applications [59].

Additionally, surface charge modulation can be combined with ligand-based targeting, e.g., antibodies or peptides, enabling both charge-assisted uptake and biological recognition for site-specific delivery. This dual-functional strategy improves overall targeting efficiency, leading to more precise drug delivery and reduced systemic side effects.

Studies investigating surface charge-dependent cellular interactions of BTNPs have demonstrated that positively charged formulations exhibit significantly higher cellular uptake compared to their neutral or negatively charged counterparts. Polyelectrolyte- coated BTNPs with positive surface charge showed enhanced internalization in cancer cells, attributed to stronger electrostatic interactions with the negatively charged cell membrane, ultimately improving the efficacy of second harmonic generation imaging-guided PDT [58,60]. This makes surface charge engineering a key design requirement for the development of next-generation BaTiO_3_-based nanotherapeutics.

### 3.7. Synergistic Drug Delivery for Cancer Cell Targeting

Systematic evaluation of the use of BTNPs synthesized via a co-precipitation method for anticancer activity against the MCF-7 human breast cancer cells, performed in [12], demonstrated pronounced cytotoxic effects in vitro. Since the BTNPs in that study were neither functionalized with targeting ligands, nor loaded with conventional chemotherapeutics, such results suggested that BTNPs possess intrinsic anticancer properties and hold potential as therapeutic agents or carriers in drug delivery platforms. Therefore, the study established important baseline evidence for biomedical applicability of BaTiO_3_ in oncological contexts. The authors attributed the enhanced cytotoxic response in part to the large surface area of the BTNPs, facilitating cellular interaction and uptake and thereby potentially improving intracellular localization of bioactive agents.

However, a major approach in nanomedicine for the enhanced delivery specificity is by the active targeting, which involves decorating the surface of nanoparticles with biomolecular ligands capable of recognizing and binding to the specific receptors expressed on the diseased cells. BTNPs are particularly well-suited for this strategy due to their chemically accessible surface, which allows easy functionalization with ligands such as antibodies, aptamers, folic acid, or transferrin [61]. Functionalization with transferrin, for instance, enabled BTNPs to target the transferrin receptor (TfR), commonly overexpressed on the surface of various cancer cells, including glioblastoma multiforme (GBM). This active targeting approach could enhance selective cellular uptake and promote localized release of therapeutic agents at the tumor site, significantly reducing off-target effects [43].

The conjugation of BTNPs with folic acid (FA) can also significantly enhance their accumulation at tumor sites, as the folate receptors are commonly overexpressed on many cancer cell types, including ovarian, breast, and lung cancers [62,63]. In [62], folic acid- functionalized PEGylated niosomes, co-encapsulating cisplatin and doxorubicin, exhibited enhanced anticancer activity in vitro and in vivo. This co-delivery system demonstrated synergistic drug interactions and permitted dose reduction while maintaining high cytotoxicity against cancer cells. In [63], a folate receptor-targeting dual-therapeutic (photothermal and chemotherapy) core–shell nanoparticle (CSNP) exhibiting a molybdenum disulfide core with a barium titanate shell (MoS2@BT) has been used to improve therapeutic efficacy against triple-negative breast cancer (TNBC) MDA-MB-231 cells. Such systems represent a strategic advancement in cancer therapy by integrating targeting selectivity and specificity, enhanced drug solubility, and stimulus-responsive behavior, all of which contribute to a higher therapeutic index and improved patient outcomes.

### 3.8. Integration into the Multifunctional Platforms

A particularly important feature of BaTiO_3_, from the viewpoint of smart drug delivery systems, is its modularity. Namely, possessing a multitude of responsive mechanisms (pH, temperature, biochemical recognition…), BTNPs can be customized to enable personalized medicine, i.e., to match the drug delivery profile to the unique pathophysiological features of the patient’s disease. In that way, therapeutic efficacy can be maximized, and the risk of adverse effects substantially reduced, aligning with modern clinical demands for safety, efficacy, and specificity. BaTiO_3_ can be embedded into micelles, liposomes, nanogels, or porous scaffolds, depending on the planned delivery route and therapeutic goal. For example, BaTiO_3_-loaded liposomes can combine passive tumor targeting via the enhanced permeability and retention (EPR) effect with active stimulus-responsive drug release, while the BaTiO_3_-hybrid hydrogels hold promise in localized drug delivery to post-surgical wounds or inflammatory tissues [44,64,65].

**Figure 4:**
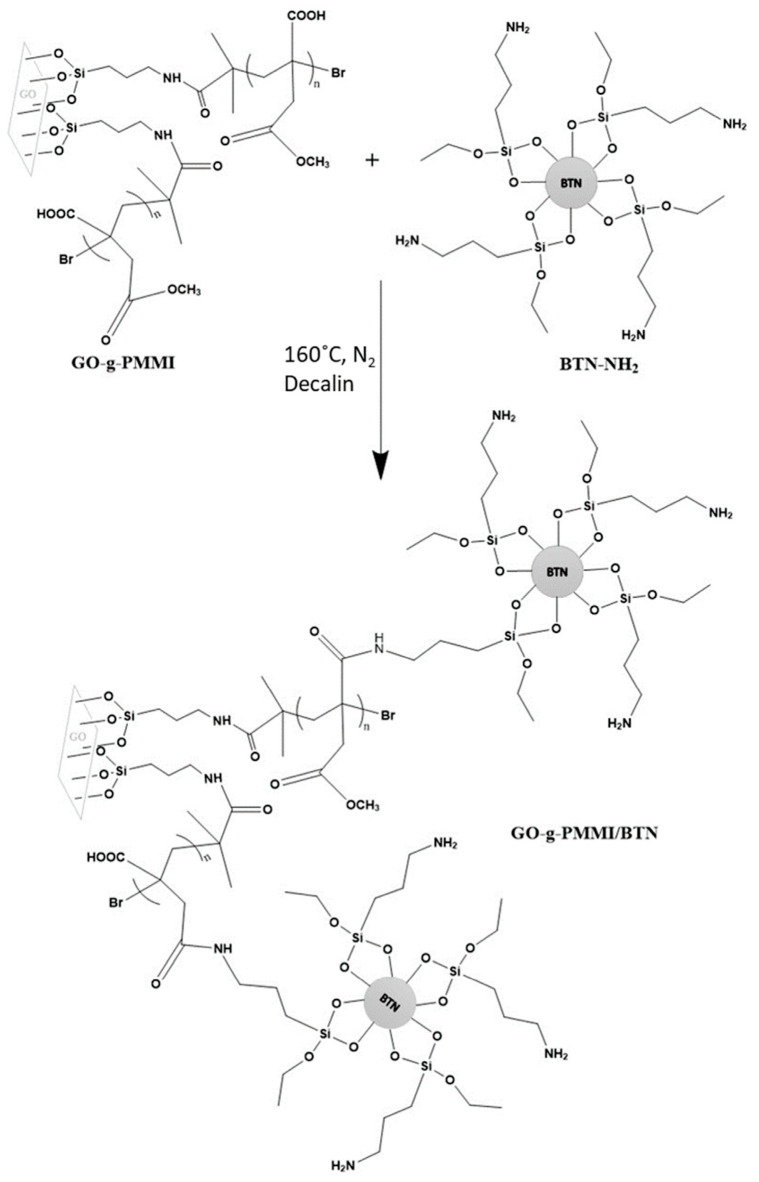
Proposed reaction scheme between silanized barium titanate nanoparticles (BTN-NH2) and GO-g-PMMI to yield hybrid GO-g-PMMI/BTN, reproduced from [66], licenced under a Creative Commons Attribution.

The integration of BTNPs into multifunctional platforms has garnered significant interest due to their unique piezoelectric properties, which can enable controlled drug delivery. On the other side, composite materials can provide enhanced therapeutic options. The synthesis of BaTiO_3_–GO (graphene oxide) nanocomposites, which leverage the large surface area and functional groups of GO to improve drug loading capacity and targeting precision, has been shown as very promising. The piezoelectric BaTiO_3_ properties enable the responsiveness of these composites to the external stimuli, e.g., ultrasound, facilitating controlled drug release, whereas the conductive nature of GO enhances the photothermal and photodynamic properties of the nanocarriers. Cuenca-Bracamonte et al. fabricated the polyetherimide-based nanocomposites filled with reduced GO and GO–BaTiO_3_ hybrid nanoparticles, as schematically represented in Figure 4 [66].

The improved electrical properties of these composites suggested their potential application in biomedical fields, including the targeted drug delivery systems. These multifunctional platforms can be further functionalized with targeting agents, such as antibodies or peptides, to achieve the site-specific delivery.

Recent research has highlighted the potential of piezoelectric BaTiO_3_ coatings to modulate immune responses through the inhibition of MAPK/JNK signaling and the activation of oxidative phosphorylation (OXPHOS) pathways [67]. A piezoelectric scaffold was prepared by hydrothermal synthesis of a uniform BaTiO_3_ layer on 3D printed Ti6Al4V scaffold (BT/Ti). In vitro results demonstrated that the piezoelectric effects promoted M2 polarization of macrophages and immunoregulatory osteogenesis of MC-3T3 osteoblasts. In a subcutaneous implantation model, higher proportion of macrophages was observed under low intensity pulsed ultrasound (LIPUS) stimulation. The study provided a promising method for regulating the immune microenvironment and enhancing bone regeneration. The findings, demonstrated here for bone regeneration models, open promising avenues for cancer immunomodulation as well, particularly in enhancing the anti-inflammatory phenotype of tumor-associated macrophages (M2 to M1 reprogramming) [67].

Finally, magnetic guidance of drug nanocarriers has attracted attention for smart drug delivery, mainly aimed at neurological and cardiovascular applications [68,69]. In [69], cobalt ferrite–barium titanate (CoFe_2_O_4_–BaTiO_3_) core–shell magnetoelectric nanoparticles have been investigated. Orthogonal Helmholtz coils have been used to induce the guiding magnetic fields on the core–shell nanoparticles. Strong coupling between magnetostrictive and piezoelectric core–shell, using ferromagnetic and ferroelectric materials allows scope for wireless control in future clinical applications.

## 4. Critical Comparison of BaTiO_3_ Nanocarriers with Conventional Drug Delivery Platforms

Conventional nanocarriers such as liposomes, polymeric nanoparticles, dendrimers, and mesoporous silica particles have significantly advanced the field of nanomedicine and have provided valuable clinical outcomes. Liposomes, in particular, represent the most clinically established systems, with several FDA- and EMA-approved formulations currently on the market. Their advantages include excellent biocompatibility, the ability to encapsulate both hydrophilic and hydrophobic drugs, and tunable surface modifications for targeted delivery. However, liposomes often suffer from drawbacks such as limited stability, aggregation, and drug leakage during storage and circulation. Moreover, their release mechanisms are largely passive, relying on diffusion or membrane destabilization, which restricts the precision of spatiotemporal drug release [70].

Dendrimers, another widely investigated class of carriers, exhibit highly controlled size and monodispersity, together with versatile surface functionality that allows the attachment of drugs, targeting ligands, or imaging probes. Their branched, tree-like architecture provides considerable design flexibility. Despite these advantages, dendrimers are limited by dose-dependent toxicity, challenges in large-scale production, and the high costs associated with their synthesis, which have so far prevented their widespread clinical adoption [71].

Mesoporous silica nanoparticles are attractive due to their large surface area, tunable pore size, and high drug-loading capacity. They enable controlled drug release and can be surface-modified for targeted therapy. Nevertheless, concerns remain regarding their long-term biodegradability and potential for chronic accumulation in vivo, which has hindered their regulatory approval [72].

In contrast, BaTiO_3_ (BT) nanocarriers represent an emerging inorganic platform with unique multifunctional properties that distinguish them from these conventional systems. BT nanoparticles exhibit ferroelectric, piezoelectric, and pyroelectric characteristics, which allow them to convert external physical stimuli such as ultrasound, mechanical stress, or temperature gradients into localized electrical potentials. This active response can be exploited to achieve precise, externally controlled, and spatiotemporal release of therapeutic agents—an advantage that is not readily attainable with traditional carriers [73]. Moreover, BaTiO_3_ is chemically stable and biocompatible, enabling long-term use in biological environments without the degradation that often limits organic carriers.

Another distinctive advantage of BaTiO_3_ nanocarriers lies in their structural tunability through doping with rare-earth ions (e.g., Ho^3+^, Er^3+^, Gd^3+^). Such modifications introduce additional functionalities, including magnetic resonance contrast or optical responsiveness, which make them suitable for theranostic applications where therapeutic and diagnostic modalities are combined within a single nanosystem. Furthermore, surface functionalization strategies such as PEGylation or SiO_2_ coating can reduce cytotoxicity, improve colloidal stability, and enhance targeting capabilities, thereby increasing therapeutic efficacy and safety.

Despite these promising features, BaTiO_3_ nanocarriers also face important challenges that must be addressed before clinical translation. Their non-biodegradable nature raises concerns regarding long-term retention in the body, necessitating systematic pharmacokinetic, biodistribution, and chronic toxicity studies. Compared to liposomes and dendrimers, which already have substantial preclinical and clinical data, BT nanoparticles remain at an earlier stage of investigation, with limited in vivo validation.

Taken together, BaTiO_3_ nanocarriers offer a novel and multifunctional alternative to conventional platforms, overcoming many of their limitations while introducing new challenges related to biodegradability and regulatory acceptance. This critical comparison underscores their potential as next-generation smart nanocarriers, while also highlighting the need for comprehensive biosafety evaluation and translational studies.

## 5. The Advantages of BaTiO_3_ and Comparison with Other Nanocarriers

### 5.1. Biocompatibility and Low Cytotoxicity

**Figure 5:**
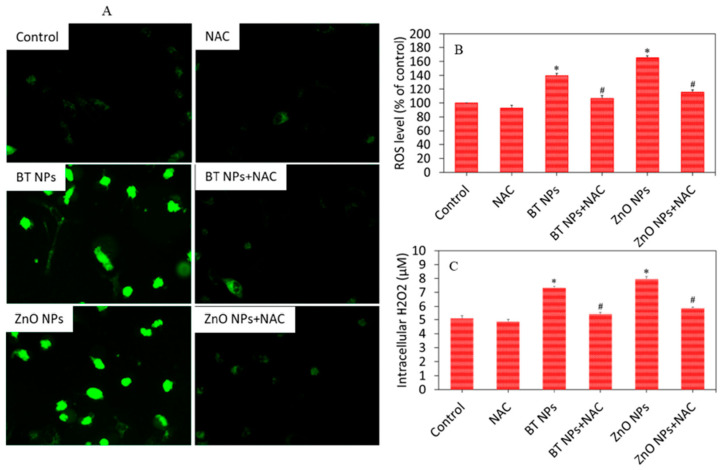
Pro-oxidant levels of A549 cells exposed for 24 h to 50 μg/mL of BT NPs with or without NAC (2 mM). ZnO NPs (25 μg/mL for 24 h) were used as a positive control. (**A**) Fluorescent microscopic images of DCF intensity in cells (an indicator of ROS generation). (**B**) Quantitative analysis of intracellular ROS level. (**C**) Quantitative analysis of intracellular H_2_O_2_ level. Data are provided as mean ± SD of three independent experiments (*n* = 3). * Indicates statistically significant difference from the control group (*p* < 0.05). # Indicates that NAC effectively abrogated the effect of BT NPs or ZnO NPs, reproduced from [74], licenced under a Creative Commons Attribution.

One of the most critical requirements for clinical translation of nanoparticle-based drug delivery systems is their biocompatibility. Up to date, BTNPs have demonstrated outstanding compatibility with biological environments, making them a promising choice for a wide range of therapeutic applications. The BTNPs have demonstrated low inherent cytotoxicity and favorable interaction with cellular structures, necessary to enable safe use in both in vitro and in vivo settings [74,75]. Several studies have investigated the effects of the interaction of BTNPs with human cancerous and non-cancerous cells. It has been noticed that BTNPs decrease the cell viability in a dose- and time-dependent manner, which differs for cancerous in comparison with the non-cancerous cells. Such behavior can be attributed to altered properties of the cancerous cells In. [74,75], cytotoxicity was selectively induced in human lung carcinoma (A549) and human colorectal carcinoma (HCT-116) cells (Figure 5). At the same time, it has been found that BTNPs did not affect normal non-cancerous cells (IMR-90, HEK-293). Application of the BTNPs has been shown to simultaneously promote tumor cell apoptosis and enhance the therapeutic penetration and selectivity. The mechanisms of action in case of the sonodynamic therapy of hypoxic tumors are primarily based on the induced electric polarization, which perturbs the local tumor microenvironment in several ways. It can enhance drug permeability by increasing membrane depolarization; it disturbs ion homeostasis in tumor cells, promoting apoptosis; additionally, it stimulates reactive oxygen species (ROS) production, leading to oxidative stress- induced cell death. Moreover, cancer cells can get sensitized to the co-administered chemotherapeutics and apoptosis-inducing ligands, overcoming drug resistance and enhancing therapy efficacy. However, BTNPs do not induce significant toxicity in normal human cells when properly engineered. They are well-tolerated by epithelial, fibroblast, and endothelial cell lines, even at moderate concentrations [37,41]. The biocompatibility of BTNPs is attributed to their chemical inertness, stability in physiological fluids, and the absence of harmful ion release. Surface functionalization of BTNPs with biocompatible polymers (such as PEG, chitosan, or dextran) or targeting ligands can further reduce cytotoxicity and improve biodistribution. These coatings can prevent undesired protein adsorption and enhance nanoparticle circulation time, which is crucial for systemic administration.

In comparison with the other nanocarriers, BTNPs have high physicochemical stability, remaining structurally intact in physiological environments. In contrast to that, liposomes often suffer from leakage, degradation, or short circulation half-life, whereas dendrimers in some occasions induce cytotoxicity due to their high surface reactivity and generation-dependent accumulation [76]. Surface-modified BTNPs have emerged as promising candidates for targeted biomedical applications due to their favorable physicochemical properties and excellent biocompatibility. As demonstrated in [7], the conjugation of specific antibodies to the surface of BTNPs enables selective binding to target cell populations without inducing significant cytotoxicity or off-target effects. This approach not only enhances therapeutic precision, but also minimizes systemic exposure, allowing for long-term use in vivo. Moreover, the study confirmed that antibody-functionalized BTNPs maintained colloidal stability and bioactivity under physiological conditions, supporting their potential in cell-specific drug delivery, diagnostic imaging, and implantable biomedical devices. The versatility of surface-engineered BTNPs makes them suitable as biocompatible platforms for sustained and localized therapeutic interventions [7].

Nonetheless, while BaTiO_3_ is generally a safe nanomaterial, it must be noted that, as any other nanoparticles, it could elicit immunological responses under certain conditions [77,78]. Improperly designed particles, exhibiting high surface reactivity or surface charge, can produce inflammation, oxidative stress, or even immune-mediated apoptosis. The immune system derangement can lead to increases in incidence of autoimmune, allergic, and even neoplastic diseases [77]. Therefore, careful optimization of size, surface charge, and colloidal stability is essential to minimize immunotoxicity. Recent findings indicate that surface-engineered BaTiO_3_ nanoparticles can suppress pro-inflammatory signaling, making them viable for long-term use in chronic therapies, implantable systems, and targeted delivery of sensitive therapeutics such as proteins or RNA-based drugs. Only by balancing safety and functionality, BaTiO_3_ nanocarriers can serve as a highly adaptable and patient-friendly platform for modern drug delivery applications.

### 5.2. Drug Loading Capacity, Modifiability, and Multifunctionality

The BaTiO_3_-based nanomaterials exhibit several distinct advantages over widely used nanocarriers such as liposomes, dendrimers, and polymeric nanoparticles, owing to their multifunctionality, piezoelectric responsiveness, and structural stability. They can serve multiple roles in combined therapeutic strategies, including drug delivery, photothermal therapy (PTT), photodynamic therapy (PDT), electrostimulation-enhanced therapy, etc. Such theranostic capability is rarely matched by traditional carriers, which are usually designed for single-function applications [54,79].

In comparison with the conventional nanocarriers, such as liposomes, limited by the internal aqueous volume of their lipid bilayer, and dendrimers, whose branched architecture can restrict payload release and influence release kinetics, BaTiO_3_-based nanomaterials exhibit a notably higher drug loading capacity. The high surface area-to-volume ratio of inorganic nanoparticles, such as barium titanate, enables efficient drug adsorption, covalent conjugation, or surface immobilization of therapeutic agents. This structural advantage allows for dense drug packing and release profile tuning, offering enhanced flexibility, stability, and overall efficacy in nanomedicine-based drug delivery [76].

Unlike conventional nanocarriers (liposomes, dendrimers), BTNPs exhibit intrinsic piezoelectric properties, converting mechanical stimuli, such as ultrasound and pressure, into the localized electric fields. As highlighted in [79], such materials hold significant promise for biomedical applications due to their ability to transduce physical energy into biologically effective signals. In the context of drug delivery, this allows for externally controlled and spatially confined drug release, offering a distinct advantage over passive carriers. Particularly relevant for sonodynamic therapy and other stimulus-responsive strategies, precise modulation of treatment location and timing is essential for maximizing the therapeutic efficacy while minimizing the off-target effects [79].

Although mesoporous silica nanoparticles (MSNs) exhibit an exceptionally high specific surface area (often between 500–1000 m^2^/g), which facilitates the loading of substantial amounts of therapeutic agents, their drug release mechanism is typically passive, relying on diffusion or pH/temperature changes. This limits their applicability in precision therapies where spatiotemporal control is essential. Additional functionalization strategies, such as stimuli-sensitive gates or enzyme-cleavable linkers, are often required to enhance responsiveness, which may compromise the system’s overall stability and complicate the synthesis procedures.

In contrast, BaTiO_3_ nanocarriers represent an emerging class of stimuli-responsive systems that can deliver drugs in a precisely controlled manner through ultrasound- triggered release, enabled by their inherent piezoelectric properties. Upon exposure to low-frequency ultrasound, BaTiO_3_ nanoparticles generate local electric fields that induce changes in their surrounding environment (ROS generation, structural reconfiguration, etc.), leading to the controlled release of loaded drugs. This mechanism allows spatiotemporal therapeutic control, reducing systemic toxicity and improving therapeutic index in targeted tissues.

Liposomal carriers, though widely used in clinical settings due to their biocompatibility and dual encapsulation capability (both hydrophilic and hydrophobic drugs), lack autonomous responsiveness to physical stimuli such as ultrasound or electric fields. They primarily rely on passive accumulation (EPR effect) or ligand-mediated targeting and do not possess intrinsic electromechanical functionality.

A recent study by Kaixi Cui et al. [80], published in Journal of Functional Biomaterials, demonstrated an ultrasound-responsive BaTiO_3_-hydrogel hybrid system for on-demand drug release, wherein piezocatalytically generated ROS under ultrasound irradiation facilitated the controlled therapeutic release. This approach represents a significant advancement over traditional nanocarrier systems, offering precise control, enhanced safety, and therapeutic efficacy, even with the lower drug-loading capacity.

Taken together, these findings position BaTiO_3_ nanocarriers as advanced platforms for smart, stimulus-responsive drug delivery, particularly suited for applications requiring localized therapeutic activation, such as solid tumors or deep-tissue inflammatory environments.

Successful synthesis of BTNPs functionalized with thiophene-based polymers, using a “grafting-from” strategy, has been reported in [81]. The presented method involved surface-initiated RAFT polymerization carried out in a controlled (“living”) fashion, enabling the covalent attachment of polymer chains directly onto the surfaces of nanoparticles. Thereby, the compatibility of BTNPs with the organic polymer matrices resulted, promoting uniform dispersion, and preventing agglomeration. The obtained nanocomposites exhibited high dielectric permittivity and low dielectric loss across a wide frequency range. The surface engineering approach played a critical role in optimizing the dielectric properties of the resulting nanocomposites. It could be extended to incorporate other π-conjugated polarizable polymer shells and different inorganic nanoparticles, enabling the development of a broad range of multifunctional materials. The strategy of functionalizing BTNPs with thiophene (or similar) polymers holds strong potential for biomedical applications, as it can provide enhanced stability in biological environments, controlled interaction with cells, and a possibility to customize nanoparticles for drug delivery, bioimaging, and theranostics, by conjugating ligands, drugs, and bioactive molecules [81].

The use of BaTiO_3_ in SDDS based on catalysis by stimuli such as pH, temperature, or enzymes can help in minimizing systemic toxicity and improving therapeutic precision. The pH responsiveness of BTNPs is particularly relevant in tumor microenvironments, where pH levels are more acidic than in healthy tissue. BTNPs can ensure the site-specific delivery by releasing drugs only under such acidic conditions [82]. Piezoelectric properties enable ultrasound-triggered release, whereby external mechanical stimuli generate localized electric fields that destabilize the drug–carrier interface, allowing for spatio- temporal control over the drug liberation [82]. BTNPs can also be engineered to interact dynamically with the physiological environment. Not only that these materials generate bioelectric signals under mechanical stimulation, promoting tissue regeneration, but also offer versatile platforms for functionalization with stimuli-responsive molecules. When modified with enzyme-sensitive linkers, BaTiO_3_-based systems can achieve site-specific drug release in response to enzymes overexpressed in inflamed or cancerous tissues. This mechanism enables a dual-function strategy, i.e., combining physical responsiveness with biochemical specificity, thereby enhancing the precision and therapeutic efficacy of nanomedicine in regenerative and oncological applications [83].

On the other side, integration of BaTiO_3_ with polymers, metals, etc., leads to the formation of multifunctional nanocarriers, which can simultaneously enable therapeutic, imaging, and diagnostic capabilities [54]. Oxygen-vacancy-engineered BaTiO_3_ (BTO-Ov) nanoparticles exhibit enhanced near-infrared (NIR) absorption and narrowed bandgaps, significantly improving their photothermal and photodynamic performance under single NIR irradiation. The engineered defects, particularly Ti^3+^ centers, enable catalytic (peroxidase-like) activity, promoting reactive oxygen species (ROS) generation in the tumor microenvironment. Incorporation of these nanoparticles into the composite systems, e.g., those combined with noble metals or biocompatible coatings, colloidal stability can be improved, as well as tumor targeting capability, and stimuli-responsiveness. The obtained hybrid nanostructures can simultaneously execute photothermal therapy (PTT), photodynamic therapy (PDT), and catalytic therapy, leading to enhanced tumor ablation through synergistic mechanisms. Confocal laser scanning microscopy (CLSM) images confirmed elevated intracellular ROS levels and reduced glutathione (GSH) in HeLa cells, indicative of oxidative stress-induced apoptosis [54]. Furthermore, in vivo assessments demonstrated significant tumor regression in treated mice, supported by thermal imaging, reduced tumor volume and mass. In [54], an integrated theranostic approach has been presented with substantial potential in cancer therapy and regenerative medicine.

### 5.3. Research Challenges and Future Directions

Despite the growing promise of barium titanate (BaTiO_3_) nanocarriers in drug delivery and biomedical applications, multiple challenges must be addressed to enable clinical translation. As emphasized in [84], complex biodistribution profiles and potential long-term toxicity of metal-based nanoparticles are major concerns to be taken into account. Systemic administration of BTNPs comes with the risk of potentially accumulating in off-target organs such as the liver, kidneys, and lungs, triggering unintended biological responses. For example, hepatic accumulation has been linked to pro-inflammatory signaling and compromised biocompatibility, raising safety concerns for chronic or repeated exposures. The permeability across the blood–brain barrier (BBB) is limited for many nanocarriers, including the BaTiO_3_, which poses a significant impediment for safe application in neurotherapeutics. The increase in systemic doses could produce additional risks for the organ toxicity. These issues highlight the necessity of advanced surface engineering and rigorous biosafety assessments to mitigate toxicity and optimize therapeutic outcomes [84].

While numerous studies reported low acute toxicity and favorable biocompatibility, toxicity problems were observed with chronic exposures, high dosages, or inadequate surface modification. As highlighted by Manke et al. [85], nanoparticles can trigger oxidative stress through excessive generation of reactive oxygen species (ROS), leading to lipid peroxidation, DNA damage, and mitochondrial dysfunction. Such mechanisms are often accompanied by the release of pro-inflammatory cytokines and activation of immune pathways [77]. Further long-term toxicological assessments are needed with the focus on immunotoxicity, inflammation, and ROS-mediated injury.

In addition, the long-term stability of BaTiO_3_ nanocarriers is essential for ensuring their safety and sustained therapeutic performance. While BaTiO_3_ is generally structurally robust, prolonged exposure to physiological fluids (e.g., blood plasma, interstitial fluids) might result in surface degradation, loss of piezoelectric functionality, or the formation of potentially harmful by-products [86]. Further studies of aging and transformation of BaTiO_3_ under physiological conditions are pivotal to developing nanocarriers that retain their mechanical, chemical, and therapeutic integrity throughout the treatments. The upcoming research should prioritize advanced design strategies and innovative synthesis methods, in order to further improve the safety, specificity, and therapeutic potential of BTNPs.

Surface functionalization of BaTiO_3_ nanoparticles (BTNPs) is a crucial strategy to enhance their biocompatibility, circulation time, and targeting efficiency. Beyond simple physicochemical stabilization, functionalization enables active recognition of disease-related receptors and reduces off-target accumulation, thereby increasing the therapeutic index. A variety of strategies have been explored, ranging from polymer coatings to ligand conjugation, each offering specific advantages and challenges.

PEGylation (polyethylene glycol modification) remains one of the most widely used approaches for improving nanoparticle performance in vivo. PEG chains create a hydrophilic corona that reduces protein adsorption (opsonization) and minimizes clearance by the reticuloendothelial system (RES). For BTNPs, PEGylation not only prolongs blood circulation but also enhances colloidal stability in biological fluids. However, recent reports have highlighted the possibility of anti-PEG immune responses in some patients, which necessitates careful optimization of PEG chain length and density.

Targeting ligands such as antibodies, peptides, and aptamers provide active targeting capabilities by binding to overexpressed receptors on tumor or diseased cells. Antibody conjugation offers high specificity and affinity, particularly for markers like HER2 or EGFR in cancer therapy. Peptide ligands, such as RGD sequences, enable selective binding to integrins that are upregulated in tumor angiogenesis. Aptamers, short oligonucleotide ligands, present another promising approach due to their small size, low immunogenicity, and ease of synthesis. Conjugation of these biomolecules to BTNPs has been shown to improve selective uptake via receptor-mediated endocytosis while reducing systemic toxicity.

Folic acid functionalization is a particularly successful example, as folate receptors are overexpressed in many tumors but shielded on normal cells. Folic acid–conjugated BTNPs thus preferentially accumulate in cancer cells, enabling higher intracellular drug delivery efficiency and reduced off-target distribution. This strategy has already been validated in several tumor models and provides a simple, cost-effective targeting method. For example, folic acid–conjugated BTNPs have been shown to efficiently target tumor cells overexpressing the folate receptors, thereby enhancing intracellular drug delivery while reducing the systemic toxicity [67,87]. Moreover, nanostructuring BTNPs, e.g., by creating core–shell or hollow nanostructures, has been shown to improve their surface area and enhance cell-particle interactions, promoting more effective uptake and bioresponse. The catalytic behavior derived from the piezoelectric properties of BaTiO_3_ can be harnessed to produce reactive oxygen species (ROS) upon external mechanical stimulation, making these materials suitable for photothermal and photodynamic cancer therapies [87].

**Figure 6:**
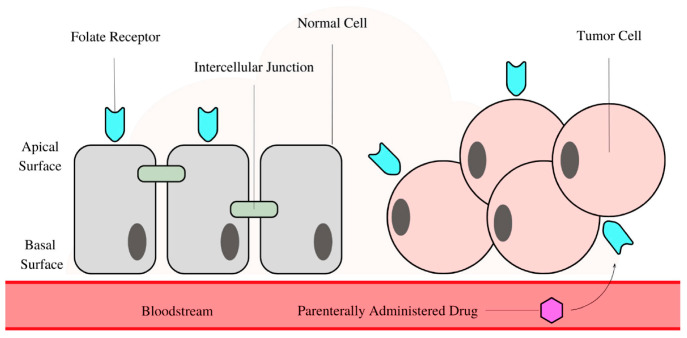
A simple diagram illustrating the folate receptor (FR) position on the normal cell apical surface compared to the FR randomly distributed over the tumor cell surface. With the intercellular junction in place, the drug is incapable to interact with FR on the apical surface, reproduced from [87], licenced under a Creative Commons Attribution.

Figure 6 illustrates the difference in folate receptor (FR) localization between normal and tumor cells, which is critical for understanding the selectivity of folic acid-conjugated BTNPs. In normal cells, FRs are confined to the apical surface, where they are physically shielded by intercellular junctions, preventing drug carriers from interacting with them. By contrast, in tumor cells, FRs are randomly distributed across the entire cell membrane, including areas not protected by tight junctions. This abnormal distribution allows folic acid- modified BTNPs to efficiently bind to and internalize into tumor cells, thereby enhancing drug delivery while minimizing off-target effects in normal tissues.

Nanostructural engineering further expands the possibilities of functionalization. The creation of core–shell or hollow nanostructures increases surface area and allows for higher ligand density or drug loading capacity. Combined with appropriate surface coatings, these architectures can significantly enhance cell–particle interactions, leading to improved nanoparticle uptake and bioresponse.

Biomimetic functionalization, such as cloaking BTNPs with natural cell membranes (e.g., red blood cells, platelets, or cancer cell membranes), represents an emerging frontier. This approach provides a “self” identity to the nanoparticles, reducing immune clearance while simultaneously imparting homotypic targeting properties. For instance, cancer cell membrane–coated BTNPs could preferentially interact with tumor tissues via adhesion molecules naturally present on the membrane.

Hybrid functionalization strategies that combine stealth polymers (such as PEG) with active ligands (such as antibodies or peptides) are also increasingly pursued. Such dual-functional designs aim to balance long circulation times with precise tumor targeting, thus optimizing therapeutic outcomes.

Another promising direction involves the integration of BaTiO_3_ with high-performance materials such as graphene nanoplatelets or transition metal dichalcogenides, resulting in multifunctional nanocomposites with superior physical, electrical, and biomedical functionalities. As demonstrated in [88,89], graphene–BaTiO_3_ polymer nanocomposites exhibit exceptional electrical conductivity, thermomechanical robustness, and electromagnetic interference (EMI) shielding. These properties arise from the strong interfacial synergy between the ferroelectric ceramic phase and the conductive graphene network, making such materials ideal for real-time sensing, implantable electronics, and structurally reinforced theranostic platforms.

In parallel, molybdenum disulfide@BaTiO_3_ (MoS_2_@BaTiO_3_) core–shell nanomedicines were developed by Murugan et al. [63], aimed at dual-modality cancer therapy. The piezoelectric and stabilizing properties of BaTiO_3_ were thereby combined with the strong photothermal conversion efficiency of MoS_2_, enabling simultaneous photothermal therapy (PTT) and chemotherapy against the triple-negative breast cancer cells. The NIR-triggered hyperthermia effect promoted drug release and deep tissue penetration, enhancing the therapeutic selectivity and minimizing the off-target toxicity. Hybrid systems like this best exemplify the potential of BaTiO_3_-based composites in the next-generation multifunctional platforms for targeted therapy, real-time diagnostics, and integrated biomedical engineering.

While showing promise in the preclinical studies, the transition of BTNPs to clinical applications faces both the significant regulatory and serious practical challenges. Firstly, the absence of harmonized and standardized protocols for evaluating the toxicity, biocompatibility, and biodistribution of BaTiO_3_ nanomaterials, slows down their clinical translation. It is essential to be able to understand and precisely describe interactions of such nanomaterials with biological systems, particularly in terms of long-term stability, clearance mechanisms, and potential immune responses. Comprehensive preclinical assessments must be able to identify any risks associated with accumulation, inflammation, or cytotoxicity of these agents. Regulatory agencies such as the U.S. Food and Drug Administration (FDA) and the European Medicines Agency (EMA) have emphasized the necessity for detailed characterization and evaluation of safety profiles of nanomaterials used in medical and pharmaceutical applications. For instance, the FDA’s guidance on Drug Products, Including Biological Products, That Contain Nanomaterials, outlines specific expectations regarding physicochemical characterization, in vitro and in vivo testing, and risk-based assessment strategies to ensure patient safety and therapeutic efficacy prior to the approval of clinical trials or market authorization [90].

Active collaboration with the regulatory bodies is vital to navigate the complex approval landscape. Engaging with agencies like the FDA and EMA early in the development process can guide necessary studies and documentation, facilitating a smoother path to clinical approval. Educational initiatives aimed at informing regulators about the unique properties and benefits of BaTiO_3_ nanocarriers could aid in aligning expectations and requirements [90,91,92].

Regarding the manufacturing and quality control of these products, the manufacturing processes for BaTiO_3_-based and other nanocarriers must adhere to the stringent Good Manufacturing Practices (GMP) to ensure consistency and high quality across production batches. Any variations in particle size, shape, or surface chemistry could significantly impact the efficacy and safety of the nanocarriers, potentially leading to clinical failures. Robust quality control measures are essential to maintaining the integrity of the nanomaterials throughout the production process [93].

Finally, regarding the commercialization and cost considerations, the high manufacturing costs associated with BaTiO_3_ nanocarriers present a significant barrier to widespread adoption. Developing cost-effective synthesis methods and scalable production techniques is essential to making the production of these nanocarriers economically viable. Furthermore, establishing clear clinical guidelines and demonstrating cost–benefit advantages over the existing therapies can enhance the attractiveness of BaTiO_3_-based treatments in the healthcare market [94].

### 5.4. Biosafety, Biodistribution, and Clinical Translation Potential of BTNPs

Despite the growing promise of barium titanate (BaTiO_3_) nanocarriers in drug delivery and biomedical applications, multiple challenges must be addressed to enable clinical translation. As emphasized in [83], complex biodistribution profiles and potential long-term toxicity of metal-based nanoparticles are major concerns to be taken into account. Systemic administration of BTNPs comes with the risk of potentially accumulating in off-target organs such as the liver, kidneys, and lungs, triggering unintended biological responses. For example, hepatic accumulation has been linked to pro-inflammatory signaling and compromised biocompatibility, raising safety concerns for chronic or repeated exposures. The permeability across the blood–brain barrier (BBB) is limited for many nanocarriers, including the BaTiO_3_, which poses a significant impediment for safe application in neurotherapeutics. The increase in systemic doses could produce additional risks for the organ toxicity. These issues highlight the necessity of advanced surface engineering and rigorous biosafety assessments to mitigate toxicity and optimize therapeutic outcomes [83].

Although in vitro studies on barium titanate nanoparticles (BTNPs) in terms of cellular compatibility and functionalization are well established, data on their in vivo behavior, including long-term toxicity, biodistribution, and immune response, are only recently emerging. Despite the limited number of available studies, several contemporary reports provide encouraging insights that support the translational potential of BTNPs. An important study by Yoon et al. (2020) [95] investigated BTNPs in the context of Tumor-Treating Fields (TTFields) in a murine breast cancer model. No signs of systemic immunotoxicity were observed after several weeks of treatment, and pro-inflammatory cytokine levels (TNF-α, IL-6) remained stable. Breast cancer cell lines MCF-7 and BT-549 showed high viability even at concentrations up to 100 μg/mL, indicating good biological tolerance and absence of acute toxicity.

Ahamed et al. (2020) [74] demonstrated that BTNPs induce selective cytotoxicity towards A549 lung carcinoma cells via oxidative stress mechanisms, while maintaining viability in normal fibroblasts (IMR-90). Mechanistic studies revealed mitochondrial dysfunction, caspase activation, increased ROS and H_2_O_2_ levels, and reduced antioxidant capacity, all of which were attenuated by N-acetylcysteine pre-treatment, confirming the oxidative stress pathway.

Candito et al. (2022) [96] systematically evaluated the effects of BTNPs on PC12 neuronal cells. No morphological or viability-related toxicity was observed, while enhanced neurite outgrowth and differentiation were noted. ROS levels and cytochrome c distribution remained unchanged, confirming the absence of oxidative stress or mitochondrial impairment.

Available short-term data (7–14 days) indicate that BTNPs primarily undergo hepatobiliary clearance, with accumulation in the liver and spleen, consistent with particles in the 50–200 nm range, which are efficiently recognized by the reticuloendothelial system (RES). No signs of hepatotoxicity or nephrotoxicity were reported; histological evaluations confirmed preserved tissue architecture, and serum markers (ALT, AST, creatinine) remained within reference values.

These findings highlight a promising safety profile of BTNPs in therapeutic and neuroregenerative contexts. Nevertheless, long-term in vivo studies (≥30 days) remain essential to assess cumulative distribution, elimination, and immune responses under repeated dosing conditions. We emphasize that these gaps do not diminish the biomedical potential of BTNPs, but rather define a strategic direction for future research—a point explicitly addressed in the revised manuscript.

It is important to recognize the limitations of the current body of in vitro and in vivo studies on BaTiO_3_ nanocarriers, as these constraints significantly affect the depth and reliability of conclusions that can be drawn regarding their long-term biosafety. Most of the available evidence originates from short-term cellular assays, often performed on a narrow selection of immortalized cell lines, or from preliminary animal studies with restricted scope and duration. While these studies provide valuable insights into acute cytotoxicity, oxidative stress, and short-term biodistribution, they are insufficient to capture the complexity of chronic exposure scenarios that are critical for translational success. Critical gaps remain regarding the potential for long-term accumulation of BaTiO_3_ nanoparticles within the reticuloendothelial system, their interaction with phagocytic cells, and the risk of delayed or cumulative toxicity. Similarly, little is known about possible effects on immune homeostasis, low-grade inflammation, or subtle alterations in the function of vital organs such as the liver, kidneys, lungs, and spleen.

In particular, systematic 90-day and longer-term toxicological investigations are lacking, despite being considered a gold standard under FDA, EMA, and ICH regulatory guidelines for evaluating the safety of nanomaterials. Such studies must encompass pharmacokinetics, quantitative biodistribution analyses, histopathological evaluation, and assessment of potential genotoxic and immunotoxic effects. Long-term data are also essential to determine whether repeated administration of BaTiO_3_ nanocarriers leads to cumulative organ burden or whether compensatory clearance mechanisms prevent their harmful accumulation. Moreover, the absence of chronic exposure models limits our ability to understand how these nanocarriers behave in diseased states, such as cancer, metabolic disorders, or immunological dysfunction, where nanoparticle clearance pathways may be significantly altered.

Addressing these knowledge gaps through rigorous, standardized, and longitudinal studies is therefore indispensable. Only by generating robust long-term datasets can the scientific community establish a comprehensive safety profile and provide the necessary evidence base for regulatory approval. Closing these gaps will be crucial not only for validating the clinical translation potential of BaTiO_3_ nanocarriers, but also for positioning them as reliable candidates in the next generation of intelligent theranostic drug delivery systems.

### 5.5. Clinical Translation and Regulatory Challenges of BaTiO_3_ Nanocarriers

The translation of BaTiO_3_ nanocarriers into the clinical context requires an integrated assessment of quality, safety, and efficacy, in strict alignment with FDA/EMA/ICH guidelines as well as good laboratory and manufacturing practices. In the early stage, the regulatory focus is on the standardization of synthesis and comprehensive characterization: control of particle size (<200 nm for systemic application), crystal phase (with a preference for the tetragonal modification due to pronounced piezoelectric properties), morphology and zeta potential, as well as chemical purity (residual alkoxides, halides, surfactants) and dopant stability. Validated methods (TEM, DLS, XRD/FTIR, zeta potential, ICP-MS) must be integrated into a GLP-compliant “assay cascade,” including immunocompatibility evaluation (cytokine release, complement activation) according to the established NCI-NCL protocols [90,91,97,98,99].

Since BT nanoparticles are inorganic and non-biodegradable, their pharmacokinetics rely on physiological clearance, with the RES/mononuclear phagocyte system favoring sequestration in the liver and spleen; hepatobiliary elimination and long-term retention are frequently observed for particles in the 50–200 nm range. Unlike renally clearable nanostructures (HD ≲ 5.5–6 nm), typical BTNPs will not be efficiently filtered through the glomerular barrier, which further underscores the need for long-term (>90 days) in vivo studies of biodistribution, retention, and elimination, ideally with radio-/element-labeled formulations and tissue quantification.

Toxicological assessment must encompass multilayered in vitro and in vivo protocols: cytotoxicity (MTT/LDH), oxidative stress (ROS, SOD, GSH), mitochondrial dysfunction (JC-1, caspases), genotoxicity (Comet assay), as well as acute, subacute, and chronic in vivo studies in at least two rodent species with hematology, serum biochemistry (ALT/AST/creatinine), and histopathology. For BTNPs, Ba^2+^ leaching and surface engineering (e.g., SiO_2_/PEG coating) are particularly relevant, as inadequate surface passivation can induce ROS-mediated cytotoxicity, whereas protective coatings significantly mitigate these effects. Upon doping with rare-earth ions (e.g., Gd^3+^, Ho^3+^, Er^3+^), both piezo/opto response and biosafety profiles are altered; since literature reports potential ROS-related adverse effects of REEs, dose and dopant stability must be carefully controlled.

For BT-based piezo-theranostic platforms (ultrasound-activated “sonosensitizer”/piezocatalytic systems), growing in vivo evidence supports their therapeutic efficacy and functional biosafety, particularly when nanoparticles are ultrasmall and PEGylated. The generation of localized piezoelectric potentials and ROS/O_2_ under US stimulation allows synergistic effects with chemotherapy and immunomodulation. For clinical translation, however, reproducibility of the piezo-response (phase composition, domain anisotropy) and clear separation of therapeutic outcomes from potential nanoparticle burden in reticuloendothelial reservoirs are crucial.

The therapeutic relevance of multimodal systems that combine photothermal therapy (PTT), photodynamic therapy (PDT), and sonodynamic therapy (SDT) lies in their ability to achieve synergistic effects that cannot be reached by monotherapies alone. PTT generates localized hyperthermia that sensitizes tumor cells to oxidative stress and enhances drug penetration, while PDT induces the production of cytotoxic reactive oxygen species (ROS) upon light irradiation. SDT complements these modalities by utilizing ultrasound to trigger ROS formation through piezoelectric or sonosensitizing mechanisms, offering the additional benefit of deeper tissue penetration compared to light-based strategies. When used in combination, these approaches can overcome tumor heterogeneity, minimize the likelihood of therapeutic resistance, and enable more effective tumor eradication at lower individual doses, thereby reducing systemic side effects. BaTiO_3_ nanocarriers are particularly promising as cores for such multimodal systems, as their intrinsic piezoelectric properties allow ultrasound-driven SDT, while doping and surface functionalization can impart photothermal or photodynamic responsiveness. This multifunctionality positions BTNP-based platforms as versatile theranostic agents that integrate multiple therapeutic modalities, offering precise spatiotemporal control and enhanced efficacy in the treatment of cancer and other complex diseases.

Immunological safety requires that BTNPs do not provoke unwanted cytokine surges (IL-1β/IL-6/TNF-α) or complement activation (C3a/C5a); NCL and related guidelines recommend a panel of assays (e.g., human heparinized whole blood and macrophage cell lines) to detect complement-related immunotoxicity, as well as identification of key factors influencing immune activation (composition, shape, dose, anti-PEG antibodies) [4,5]. For parenteral formulations, sterility (USP <71>/Ph. Eur. 2.6.1) and apyrogenicity are mandatory: LAL/BET testing in compliance with USP <85>/Ph. Eur. 2.6.14 (with validation of interference), while in Europe recombinant Factor C methods are increasingly accepted as alternatives [100,101].

From the production standpoint, GMP-compliant synthesis under controlled conditions (QbD/QMS; ICH Q8/Q9/Q10) and validation of every step (raw materials, batch consistency, in-process controls, stability) are prerequisites before IND/CTA filings. EMA and FDA emphasize that nanomaterials incorporated into medicinal products must meet the same quality and safety standards as conventional drugs, with additional nanomedicine-specific characterization [90,91,97,98,99].

Overall, although BT nanoparticles represent a highly promising theranostic platform, their translation into clinical practice requires a strictly controlled, long-term, and systematic validation process. Only through the integration of regulatory standards, comprehensive toxicological evaluations, immunocompatibility testing, and GMP-grade manufacturing protocols can BTNPs achieve clinical acceptability as the next generation of intelligent drug delivery systems.

## 6. Conclusions

The application of nanotechnology in medicine has transformed drug delivery strategies, enabling the development of systems for spatially and temporally controlled release of therapeutic agents. In this context, barium titanate (BaTiO_3_) nanoparticles have emerged as a particularly promising platform due to their unique combination of dielectric, ferroelectric, and piezoelectric properties, along with high biocompatibility. These features allow BTNPs not only to encapsulate and transport drugs but also to release them selectively in response to either internal physiological cues or externally applied stimuli. They can respond dynamically to the pH gradients, enzymatic activity, temperature fluctuations, and ultrasound stimulation. The BTNPs are being customized to provide targeted treatment of complex diseases such as cancer, neurological disorders, and inflammatory conditions. Precise drug delivery localization allows for the reduced systemic toxicity, as well as enhanced therapeutic efficacy.

By the integration with targeting ligands, biocompatible coatings, and functional composite elements such as gold, silver, or graphene, BaTiO_3_-based systems can be transformed into the multifunctional platforms that combine therapy, diagnostics, and real-time monitoring. BaTiO_3_-based nanostimulators have been used to enhance both the penetration of therapeutic agents across the blood–brain barrier and the intracellular delivery of anticancer drugs in glioblastoma treatment models.

Both scientific and regulatory barriers slow down the wider adoption of these systems. Future research will have to provide refined synthesis techniques, including precise control of particle size, surface charge, and crystal phase, as well as surface functionalization with polymers, targeting moieties, or enzymatically cleavable linkers in order to enhance specificity and reduce off-target effects. Long-term structural and functional stability must be ensured, to avoid compromising therapeutic performance or forming toxic by-products.

In summary, BaTiO_3_ nanocarriers represent a highly versatile and innovative class of materials with the potential to redefine how drugs are delivered and monitored in the body. Their application extends beyond traditional therapeutic delivery to include responsive, intelligent systems capable of real-time interaction with the biological environment. The coordinated research efforts, improved standardization in preclinical testing, and the development of GMP-compliant manufacturing to ensure reproducibility, scalability, and quality control, are the key elements to allow successful commercialization of BTNPs. Thereby, they could provide personalized, safe, and efficient medical treatments, which are envisioned for the era of precision medicine.

## Data Availability

No new data were created during the writing of this review article.

## Abbreviations

The following abbreviations are used in this manuscript:

BTNPs	Barium titanate (BaTiO_3_) nanoparticles
SDDS	Smart drug delivery systems
SDT	Sonodynamic therapy
LIPUS	Low intensity pulsed ultrasound
PTT	Photothermal therapy
PDT	Photodynamic therapy
BTO-Ov	Oxygen-vacancy-engineered barium titanate
NIR	Near-infrared
ROS	Reactive oxygen species
EPR	Enhanced permeability and retention
SHG	Second-harmonic generation
CLSM	Confocal laser scanning microscopy
BBB	Blood–brain barrier
GBM	Glioblastoma multiforme
TNBC	Triple-negative breast cancer
TfR	Transferrin receptor
FA	Folic acid
GSH	Glutathione
CSNP	Core–shell nanoparticle
GBT	Graphene–barium titanate nanocomposite
GO–BaTiO_3_	Graphene oxide–barium titanate nanocomposite
PMMA/PEO	Poly(methyl methacrylate)/Poly(ethylene oxide)
PEO/SF	Poly(ethylene oxide)/Silk fibroin
PHB	Polyhydroxybutyrate
PEI	Polyethyleneimine
DSPE-PEG	A lipid-polymer conjugate composed of 1,2-distearoyl-sn-glycero- 3-phosphoethanolamine (DSPE) and polyethylene glycol (PEG)
RAFT	Reversible Addition-Fragmentation chain Transfer polymerization
MAPK/JNK	Mitogen-activated protein kinase/Jun N-terminal kinase signaling pathway
OXPHOS	Oxidative phosphorylation
MLCCs	Multilayer ceramic capacitors
ML	Machine learning
FDA	U.S. Food and Drug Administration
EMA	European Medicines Agency
GMP	Good Manufacturing Practices
